# Association of Plasma and Electroencephalography Markers With Motor Subtypes of Parkinson’s Disease

**DOI:** 10.3389/fnagi.2022.911221

**Published:** 2022-07-12

**Authors:** Xiaoxia Yang, Zhen Li, Lipeng Bai, Xiao Shen, Fei Wang, Xiaoxuan Han, Rui Zhang, Zhuo Li, Jinghui Zhang, Mengmeng Dong, Yanlin Wang, Tingyu Cao, Shujun Zhao, Chunguang Chu, Chen Liu, Xiaodong Zhu

**Affiliations:** ^1^Department of Neurology, Tianjin Neurological Institute, Tianjin Medical University General Hospital, Tianjin, China; ^2^National Health Commission Key Laboratory of Hormones and Development, Tianjin Key Laboratory of Metabolic Diseases, Chu Hsien-I Memorial Hospital & Tianjin Institute Endocrinology, Tianjin Medical University, Tianjin, China; ^3^School of Electrical and Information Engineering, Tianjin University, Tianjin, China

**Keywords:** Parkinson’s disease, motor subtype, TD, PIGD, EEG, biomarker, plasma

## Abstract

**Objective:**

The aim of this study was to investigate the correlations of plasma neurodegenerative proteins and electroencephalography (EEG) dynamic functional network (DFN) parameters with disease progression in early Parkinson’s disease (PD) with different motor subtypes, including tremor-dominant (TD) and postural instability and gait disorder (PIGD).

**Methods:**

In our study, 33 patients with PD (21 TD and 12 PIGD) and 33 healthy controls (HCs) were enrolled. Plasma neurofilament light chain (NfL), α-synuclein (α-syn), total-tau (t-tau), β-amyloid 42 (Aβ42), and β-amyloid 40 (Aβ40) levels were measured using an ultrasensitive single-molecule array (Simoa) immunoassay. All the patients with PD underwent EEG quantified by DFN analysis. The motor and non-motor performances were evaluated by a series of clinical assessments. Subsequently, a correlation analysis of plasma biomarkers and EEG measures with clinical scales was conducted.

**Results:**

In the TD group, plasma NfL exhibited a significant association with MDS-UPDRS III and Montreal Cognitive Assessment (MoCA). A higher Aβ42/40 level was significantly related to a decrease in Hamilton Depression Rating Scale (HAMD) and Hamilton Anxiety Rating Scale (HAMA) in the PIGD group. In terms of the correlation between EEG characteristic parameters and clinical outcomes, trapping time (*TT*) delta was positively correlated with MDS-UPDRS III and MoCA scores in the TD group, especially in the prefrontal and frontal regions. For other non-motor symptoms, there were significant direct associations of *k*_*PLI*_ theta with HAMD and HAMA, especially in the prefrontal region, and *k*_*PLI*_ gamma was particularly correlated with Rapid Eye Movement Sleep Behavior Disorder Screening Questionnaire (RBDSQ) scores in the prefrontal, frontal, and parietal regions in the TD group. Furthermore, there was a significant positive correlation between plasma t-tau and *k*_*PLI*_, and pairwise correlations were found among plasma NfL, theta *TT*, and MoCA scores in the TD group.

**Conclusion:**

These results provide evidence that plasma neurodegenerative proteins and EEG measures have great potential in predicting the disease progression of PD subtypes, especially for the TD subtype. A combination of these two kinds of markers may have a superposition effect on monitoring and estimating the prognosis of PD subtypes and deserves further research in larger, follow-up PD cohorts.

## Introduction

Parkinson’s disease (PD) is a common chronic progressive neurodegenerative disease with a series of etiologies and clinical manifestations, and it eventually has an adverse impact on quality of life, with a range of physical, emotional, and economic consequences. The prevalence of PD is gradually increasing with age and tends toward the younger population, affecting approximately 1% of the population aged over 60 (Tysnes and Storstein, 2017). It is acknowledged that patients with PD are classified into three subtypes: tremor-dominant (TD), postural instability and gait disorder (PIGD), or indeterminate, derived from the defined and universally recognized algorithms utilizing the Movement Disorder Society Unified Parkinson’s Disease Rating Scale (MDS-UPDRS) (Stebbins et al., 2013). It is of vital importance to identify and verify reliable biomarkers that could mirror the preclinical and early stages of different PD motor subtypes, providing a reference for their correct and timely therapeutic management.

Parkinson’s disease comprises a clinically heterogeneous group of motor subtypes with diverse progression patterns, characterized by different motor symptoms and non-motor symptoms (NMSs). NMSs precede motor dysfunction sometimes by several years, including hyposmia, sleep disorders, depression, bladder dysfunction, constipation, and even fatigue (Lee et al., 2013; Pfeiffer, 2016), which virtually are more crushing and crippling than motor symptoms. The utility of accessible and objective blood-based biomarkers, particularly correlated with motor or non-motor trajectories of PD phenotypes, could achieve an appropriate and credible correspondence with clinical consequences. Currently, several biomarkers associated with neurodegeneration, such as neurofilament light chain (NfL), α-synuclein (α-syn), total-tau (t-tau), and β-amyloid 42 (Aβ42) and β-amyloid 40 (Aβ40), have been investigated as potential predictors of disease progression. There is reliable evidence that higher NfL was associated with significantly worse global cognition and MDS-UPDRS motor scores in the PIGD group (Ng et al., 2020), and the plasma concentration of Aβ42 was significantly associated with the severity of PIGD score (Ding et al., 2017). Accordingly, plasma biomarkers may be reliable tools for predicting disease severity and progression in PD subtypes.

It is acknowledged that brain activity and network vary in different patients with PD with personalized motor symptoms and NMSs. Complexity and dynamic functional connectivity (dFC) within and between cerebral regions could mirror motor and cognition organization and functional changes to some extent (Chu et al., 2020; Yi et al., 2022). Accordingly, resting-state electroencephalography (EEG) recording at the scalp composed of electric potential discrepancy not only has particular attractions compared to MRI or PET imaging, such as non-invasiveness, low cost, and wide acceptability, but also directly reflects cortical rhythms and imperceptible network changes (Gratwicke et al., 2015). Previous studies implied that different PD motor subtypes have different degeneration of subcortical/cortical pathways and structures and influence the activity of multiple cortical functional regions, such as motor, cognition, emotions, and sleep (Jiang et al., 2016; Vervoort et al., 2016b). Changes in active brain regions linked to motor/sensorimotor areas have been exhibited in different PD motor subtypes (Orcioli-Silva et al., 2021). Vervoort et al. (2016a) have shown differential connectivity alterations in neural networks and between motor and cognitive control loops that correlated with the behavioral heterogeneity in patients with TD and PIGD. Similar to plasma biomarkers mentioned above, utility resting-state EEG for brain activity feature extraction is also a reliable and easily achievable method. Hence, a combination of blood-based and EEG measurements may have a synergistic effect to explore the predictors of disease progression in early PD with different motor subtypes.

The aim of this study was to investigate the association of plasma neurodegenerative proteins (NfL, α-syn, t-tau, Aβ42, and Aβ40) and EEG signature with disease progression in early PD with different motor subtypes. In this study, we used an ultrasensitive single-molecule array (Simoa) immunoassay for plasma biomarker measurement and a novel electroencephalographic analysis technique-dynamic functional network (DFN) analysis for electroencephalographic feature extraction.

## Materials and Methods

### Study Participants

A total of 36 patients with PD were recruited from the movement disorders outpatient clinics of the General Hospital of Tianjin Medical University. All of the patients with PD fulfilled the United Kingdom Parkinson’s Disease Society Brain Bank (UKPDSBB) criteria (Hughes et al., 1992). Based on the MDS-UPDRS classification method, the ratio of the mean UPDRS tremor scores (11 items) to the mean UPDRS-PIGD scores (5 items) was used to define patients with TD (ratio ≥1.15), patients with PIGD (ratio ≤0.9), and “indeterminate” patients (ratios >0.9 and <1.15) (Stebbins et al., 2013). Patients defined as “indeterminate” were excluded from our study. Eventually, 33 patients with PD (21 TD and 12 PIGD) were enrolled in our analysis. Simultaneously, 33 age-matched healthy controls (HCs) who showed no sign of neuropsychiatric and systemic disorder were recruited from the local community during the same period. The research protocols were approved by the Ethics Committee of the General Hospital of Tianjin Medical University. All subjects provided informed written consent before entering the study.

### Clinical Evaluation

The motor symptoms of patients with PD were evaluated using the MDS-UPDRS (Goetz et al., 2008) and Hoehn and Yahr (H&Y) staging scale (Hoehn and Yahr, 1967) as measurements of clinical parkinsonian severity, which were performed during off medication, more than 12 h after the last dose of dopaminergic therapy. NMSs were examined with a series of neuropsychological assessments, including the Montreal Cognitive Assessment (MoCA), Hamilton Depression Rating Scale (HAMD), Hamilton Anxiety Rating Scale (HAMA), Non-motor Symptoms Questionnaire (NMSQ), Rapid Eye Movement Sleep Behavior Disorder Screening Questionnaire (RBDSQ), and Parkinson’s Disease Sleep Scale-2 (PDSS-2).

### Measurement of Plasma Biomarkers

A total of 10 ml of venous blood from all the participants was collected in tubes containing ethylenediaminetetraacetic acid (EDTA) and centrifuged (2,500 × *g* for 15 min) within 1 h after collection according to the recommendation by the manufacturer and previous reports. Plasma was stored in polypropylene tubes at −80°C until analysis. The Simoa NfL Advantage kits (Quanterix, Lexington, MA, United States), Neurology 3-Plex A Advantage Kit (Lot 502473), and α-syn discovery kit (Lot 502566) were used for measurement of plasma NfL, t-tau, Aβ42, Aβ40, and α-syn concentrations assayed by researchers who were blinded to the diagnosis based on manufacturer’s introductions and standard procedures.

### Electroencephalography Recording and Preprocessing

Electroencephalography was recorded for all patients with PD during off medication in an isolated low-light room at the General Hospital of Tianjin Medical University. Patients were requested to sit in a comfortable chair with their eyes closed but not fall asleep. According to the international standard 10/20 system, 19 Ag/AgCl electrodes were placed on the scalp, including channels Fp1, Fp2, F3, F4, C3, C4, P3, P4, O1, O2, F7, F8, T3, T4, T5, T6, Fz, Cz, and Pz. Meanwhile, electrooculogram (EOG), electromyogram (EMG), and electrocardiogram (ECG) signals were recorded through another four channels.

All of the EEG preprocessing was performed using the EEGLab toolbox in MATLAB software (MathWorks Inc., Natick, MA, United States). First, EEG signals were processed by a 1–45 Hz band-pass zero-phase shift filter for filtering out the 50 Hz power frequency interference to ensure that the phase information of the original signal remained unchanged. To eliminate artifacts, fast independent component analysis (FastICA) (Hyvärinen, 1999) was then conducted. The FastICA algorithm decomposed 19-channel EEG signals into ICs that are statistically independent of each other through a hybrid matrix. Subsequently, the correlation between the extracted ICs and the EOG, EMG, and ECG signals was analyzed. The IC whose absolute value of the correlation coefficient exceeds 0.5 was considered as the component that has a strong correlation with a certain artifact signal. We then zeroed out these ICs and multiplied them by the resulting mixture matrix to obtain the EEG signals with the artifacts removed. The manual screen was applied to remove noise interference signals. Finally, a band-pass finite impulse response (FIR) filter was used to filter the signals into five frequency bands: delta (1–4 Hz), theta (4–8 Hz), alpha (8–13 Hz), beta (13–30 Hz), and gamma (30–45 Hz).

### Construction of Dynamic Functional Networks

The empirical mode decomposition (EMD) method could well describe the local transient characteristics of time-varying non-linear signals. EMD could divide different windows according to the signal characteristics of different patients, facilitating the finding of information with significant differences in time-varying signals (Chen et al., 2010). The sliding window method based on EMD could determine the dynamic window length by the frequency of EEG signals of patients at different times. A previous study reported that this adaptive window method could obtain better dynamic information about cognitive function and behavior (Zhuang et al., 2020). Hence, in this study, we utilized the adaptive sliding window based on EMD to seek out the local information on dynamic brain activity in patients with PD.

Based on the data after dividing windows by the above method, we used the phase lag index (PLI) to characterize DFNs. The PLI method, proposed by Stam et al. (2007), could estimate the phase synchronization between signals. We used PLI to calculate the phase coupling degree by calculating the instantaneous phase and the asymmetry of the phase difference distribution between two time signals, and the calculation formula is as follows:


$$P\,L\,I=\left|\left\langle \text{sign}\,\left[\Delta \,\phi \,\left(t_{k}\right)\right]\right\rangle \right|,k=1,2,\ldots ,N,$$

where Δϕ(*t*_*k*_) represents phase differences calculated at different times. *PLI* ranges from 0 to 1. The larger the *PLI*, the stronger the coupling strength between signals.

### Fluctuation Analysis of Dynamic Functional Connectivity

The noise of EEG signals could also produce random fluctuations of network connection, such that the existence of dFC could not be proved only by the FC fluctuations of EEG signals collected from patients. We used alternative data to observe if the fluctuations in FC were representative of the real dFC. We represented stationary processes by using suitable alternative data to verify the authenticity of fluctuations and used the amplitude-adjusted Fourier transform (AAFT) to construct alternative data (Kugiumtzis, 2002). The AAFT method was used to generate 20 groups of alternative data corresponding to real data, and then we used the window of real data selection to partition and constructed the dFN of alternative data. By calculating the time-series standard deviations of each edge in DFN’s dynamic process of real data and alternative data, we obtained the dynamic fluctuation characteristics of brain connectivity for both. We calculated the average fluctuation of 20 sets of alternative data and the *k*_*PLI*_, which was the ratio of standard deviation between fluctuations in real data and fluctuations in alternative data. The formula is given as follows:


$$k_{P\,L\,I}\,\left(v,w\right)=\frac{\sigma \,\left(D_{t\,u\,r\,e}\,\left(v,w\right)\right)}{1/M\,\sum_{i=1}^{M}\sigma \,\left(D_{r\,a\,n\,d}\,\left(v,w\right)\right)},$$

where *D* represents the DFNs of real data and alternative data, *v* and *w* are EEG channels, and *M* is the group number of alternative data. As the ratio of fluctuation of real data to the fluctuation of substitute data, if *k*_*PLI*_ was greater than 1, then there was a real fluctuation in dynamic brain FC, and *k*_*PLI*_ could represent the magnitude of fluctuation.

### Network State Transition Analysis

#### Time-by-Time Graph

We used the form of the weighted DFNs to define time in constructing the time-by-time graph (Medaglia et al., 2015). Each node represented a different time window of DFNs, and the different edges indicated the correlations between the corresponding time windows of DFNs. We utilized the Frobenius norm for distance measurement to calculate the similarity between networks (Kurmukov et al., 2016), and the distance measure was inversely proportional to similarity. The calculation formula is as follows:


$$d_{F}\left(P\left(t_{1}\right),P\left(t_{2}\right)\right)=∥P\left(t_{1}\right)-P\left(t_{2}\right){∥}_{F},=\sqrt{\sum_{i=1}^{N}\sum_{j=1}^{N}{\left(p_{i\,j}\,\left(t_{1}\right)-p_{i\,j}\,\left(t_{2}\right)\right)}^{2}}$$

where *P* represents DFNs of different patients, *p* represents the edge of DFNs, *i* and *j* are the EEG channels, and *N* represents the total number of EEG channels. The time-by-time graph *T* of each patient was obtained, through which we could get the necessary information on the same brain state during the evolution of dynamic brain functional networks (Chen et al., 2017).

#### Recurrence Plot

Based on the time-by-time graph obtained from the patient, we determined a threshold to define the upper limit of the distance between similar networks. When the calculated distance was less than a, we concluded that the two brain functional networks were in the same state and set the edge of the time-by-time graph *T*_*ij*_ to 1. On the contrary, we set it to 0. We could get the recurrence plot (RP) using this method, and the recurrence matrix R of DFNs is as follows:


$$R_{i\,j}=\left\{ \begin{array}{c} 1,T_{i\,j}<a\\ 0,T_{i\,j}>a \end{array}\right.,$$

where *i* and *j* are the different times of the brain’s functional network, and *T*_*ij*_ is the distance measure of the functional network at two moments.

#### Recurrence Quantification Analysis

We used the recurrence quantification analysis (RQA) parameters, which were recurrence rate (RR) and trapping time (*TT*), to evaluate the degree of network state transition of DFNs (Marwan et al., 2007). *RR* indicated the number of times that a network in the same state occurred in DFNs. The *RR* is defined as follows:


$$R\,R=\frac{1}{N^{2}}\,\sum_{i,j=1}^{N}R_{i\,j},$$

where *N* represents the total number of brain functional networks at different time windows. *TT* represents the average duration of the same network state in DFNs. The larger the *TT*, the longer the same state lasts on the network. The *TT* is defined as follows:


$$T\,T=\frac{\sum_{h=h_{\min}}^{h_{\max}}h\,P\,\left(h\right)}{\sum_{h=h_{\min}}^{h_{\max}}P\,\left(h\right)},$$

where *h* was the length of the vertical line in *RP*, and *P*(*h*) is the number of vertical lines whose length is *h* in *RP*. The *P*(*h*) is calculated as follows:


$$P\,\left(h\right)=\sum_{i,j=1}^{N}\left(1-R_{i,j-1}\right)\,\left(1-R_{i,j+h}\right)\,\prod_{k=0}^{h-1}R_{i,j+k}$$

### Statistical Analysis

All data were analyzed using SPSS 22.0 (IBM, Inc., Armonk, NK, United States) and GraphPad Prism 9 (La Jolla, CA, United States). The Shapiro–Wilk test was used to examine the Gaussian distribution of our data (*p* > 0.05). The demographic and clinical characteristics were displayed as mean ± SD. Comparisons of continuous variables among different diagnostic groups were assessed using one-way analysis of variance (ANOVA) and the Mann–Whitney *U* test. A Chi-square test was used to compare categorical variables. Group comparisons of clinical assessments and marker measures were made using multiple linear regression analyses with sex, age, and disease duration at testing as covariates. The correlation among plasma neurodegenerative proteins, EEG characteristic parameters, and clinical outcomes was accessed using Spearman’s rank correlation analysis. Statistical significance was set at *p* < 0.05.

## Results

### Demographic and Clinical Characteristics

A total of 66 participants consisting of 33 patients with PD and 33 age- and sex-matched normal control subjects were enrolled in this study. The demographic and clinical characteristics of study participants are presented in Table 1. There were no significant differences in gender and age between patients with PD and HC. Similarly, in terms of disease duration, H&Y stage, MDS-UPDRS II and III scores, global cognition status-MoCA, and other non-motor scales, no statistical differences were found between the two motor phenotypes. In the PIGD group, the score of MDS-UPDRS I was significantly higher than the TD group (*p* < 0.01).

**TABLE 1:** Demographic and clinical characteristics of study participants.

Characteristics	TD (*n* = 21)	PIGD (*n* = 12)	HC (*n* = 33)	*P*-value
Male, %	47.62	50.00	51.52	0.96
Age, years	65.48 ± 6.83	63.92 ± 5.78	66.15 ± 4.75	0.74
Disease duration, years	5.38 ± 2.52	5.00 ± 1.81	NA	0.99
Hoehn and Yahr stage	1.24 ± 0.44	1.67 ± 0.49	NA	0.03
MDS-UPDRS I	2.00 ± 2.03	4.45 ± 2.70	NA	<0.01**
MDS-UPDRS II	4.21 ± 3.99	5.09 ± 5.20	NA	0.87
MDS-UPDRS III	19.71 ± 11.71	18.58 ± 11.12	NA	0.90
MoCA	26.32 ± 2.96	25.73 ± 3.74	NA	0.93
NMSQ	5.62 ± 3.79	8.42 ± 4.33	NA	0.06
RBDSQ	2.79 ± 3.61	3.27 ± 3.80	NA	0.54
HAMD	2.58 ± 3.49	5.09 ± 4.04	NA	0.06
HAMA	1.47 ± 2.17	4.91 ± 7.35	NA	0.10
PDSS-2	4.84 ± 4.51	11.91 ± 8.87	NA	0.02

*PD, Parkinson’s disease; TD, tremor-dominant; PIGD, postural instability and gait disorder; HCs, healthy controls; MoCA, Montreal Cognitive Assessment; NfL, neurofilament light chain; MDS-UPDRS, Movement Disorder Society Unified Parkinson’s Disease Rating Scale; HAMD, Hamilton Depression Rating Scale; HAMA, Hamilton Anxiety Rating Scale; NMSQ, Non-motor Symptoms Questionnaire; RBDSQ, Rapid Eye Movement Sleep Behavior Disorder Screening Questionnaire; PDSS-2, Parkinson’s Disease Sleep Scale-2; NA, not available. Data are presented as mean ± SD. The p-values were obtained from comparisons of variables between TD and PIGD using the Mann–Whitney U test. The Chi-square test was used to compare categorical variables.*

**p < 0.01.

### Plasma Biomarker Levels and Electroencephalography Characteristic Parameters in Different Groups

Plasma biomarkers and EEG characteristic parameters are summarized in Table 2. The levels of plasma Aβ42, Aβ40, Aβ42/40, and α-syn were significantly increased in patients with PD when compared to controls (Aβ42: TD vs. HC: *p* < 0.0001, PIGD vs. HC: *p* < 0.0001; Aβ40: TD vs. HC: *p* < 0.0001, PIGD vs. HC: *p* < 0.001; Aβ42/40: TD vs. HC: *p* < 0.05, PIGD vs. HC: *p* < 0.01; α-syn: TD vs. HC: *p* < 0.0001, PIGD vs. HC: *p* < 0.001), while there was no significant difference between the TD and PIGD groups, after adjustment for age, sex, and disease duration. No differences were found in the concentrations of plasma NfL and t-tau among the TD, PIGD, and control groups (NfL, *p* = 0.65; t-tau, *p* = 0.39). Similarly, there was no difference in EEG characteristic parameters (*k*_*PLI*_, *TT*, *RR*) between the TD and PIGD groups (*k*_*PLI*_, *p* = 0.49; *TT*, *p* = 0.51; *RR*, *p* = 0.59, Table 2).

**TABLE 2:** The levels of plasma biomarkers and EEG characteristic parameters in patients with PD and HCs.

	TD (*n* = 21)	PIGD (*n* = 12)	HC (*n* = 33)	*P*-Value
**Plasma biomarker**				
NfL (pg/ml)	18.01 ± 10.16	25.32 ± 22.10	15.99 ± 5.18	0.65
T-tau (pg/ml)	1.05 ± 0.57	0.87 ± 0.23	0.93 ± 0.63	0.39
Aβ42 (pg/ml)	9.93 ± 2.58	10.48 ± 2.58	5.26 ± 2.01	<0.01^a^,^b^
Aβ40 (pg/ml)	165.43 ± 48.40	162.37 ± 40.92	103.90 ± 31.00	<0.01^a^,^b^
Aβ42/40	0.06 ± 0.01	0.07 ± 0.01	0.05 ± 0.01	<0.01^a^,^b^
α-Syn (pg/ml)	49.27 ± 41.06	41.39 ± 24.37	10.36 ± 6.51	<0.01^a^,^b^
**EEG parameter**				
*K*_*PLI*_	1.91 ± 0.07	1.20 ± 0.10	NA	0.49
*TT*	16.85 ± 18.70	17.88 ± 23.77	NA	0.51
*RR*	0.34 ± 0.25	0.38 ± 0.30	NA	0.59

*PD, Parkinson’s disease; TD, tremor-dominant; PIGD, postural instability and gait disorder; HCs, healthy controls; NfL, neurofilament light chain; t-tau, total tau; Aβ42, β-amyloid 42; Aβ40, β-amyloid 40; α-syn, α-synuclein; NA, not available.*

*^a^Differences were found between TD vs. control.*

*^b^Differences were found between PIGD vs. control.*

### Plasma Biomarker Correlations With Clinical Outcomes

**FIGURE 1:**
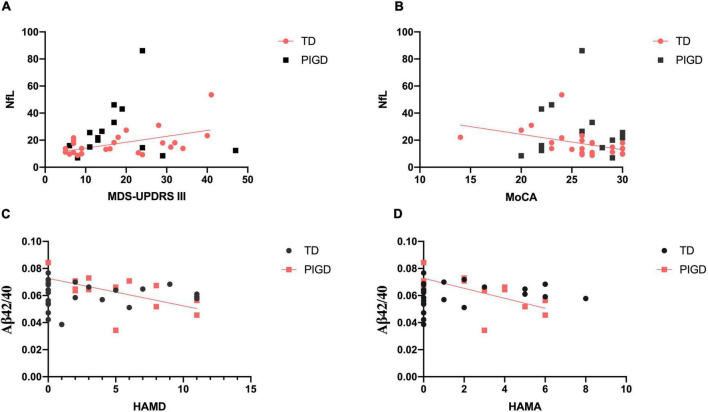
Correlations between plasma biomarkers and clinical features in patients with PD. There were significant relationships between plasma NfL and MDS-UPDRS III **(A)** and MoCA **(B)** in the TD group. Plasma Aβ42/40 level was negatively correlated with HAMD **(C)** and HAMA **(D)** in PIGD. Significant correlation (*p* < 0.05) is marked in solid red line.

For clarifying whether there was any relationship between plasma biomarkers and clinical features, we conducted the following correlation analysis. In the TD group, plasma NfL exhibited significant association with MDS-UPDRS III (*r* = 0.443, *p* < 0.05, Figure 1A) and MoCA (*r* = −0.555, *p* < 0.01, Figure 1B). Higher Aβ42/40 level significantly related to a decrease in HAMD (*r* = −0.590, *p* < 0.05, Figure 1C) and HAMA (*r* = −0.635, *p* < 0.05, Figure 1D) in the PIGD group. No relationship was found between any plasma biomarkers and NMSQ, RBDSQ, and PDSS-2 scores.

### Electroencephalography Correlations With Clinical Outcomes

#### Correlations Between Electroencephalography Signature and Motor Severity

**FIGURE 2:**
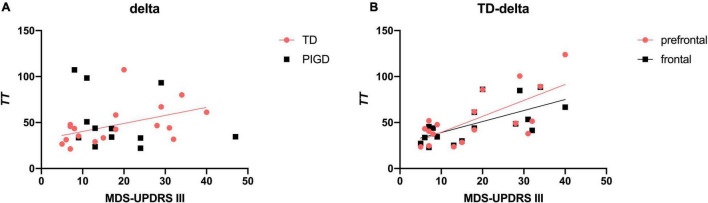
Correlation between *TT* delta and MDS-UPDRS III scores in patients with PD. *TT* delta was positively associated with MDS-UPDRS III scores **(A)**, especially in the prefrontal and frontal regions **(B)**. A significant correlation (*p* < 0.05) is marked in solid red and black lines.

We performed exploratory correlation analysis for the EEG signature as a marker for clinical features and disease progression. Meanwhile, EEG parameters were further refined into brain regions to explore the brain regions with specific changes in different motor subtypes of patients with PD with different clinical manifestations. The results suggested that *TT* delta was positively correlated with MDS-UPDRS III scores in the TD group (*r* = 0.585, *p* < 0.05, Figure 2A), especially in prefrontal (*r* = 0.638, *p* < 0.01, Figure 2B) and frontal (*r* = 0.685, *p* < 0.01) regions. No such association was found between EEG parameters and the H&Y stage.

#### Correlations Between Electroencephalography Signature and Cognition

**FIGURE 3:**
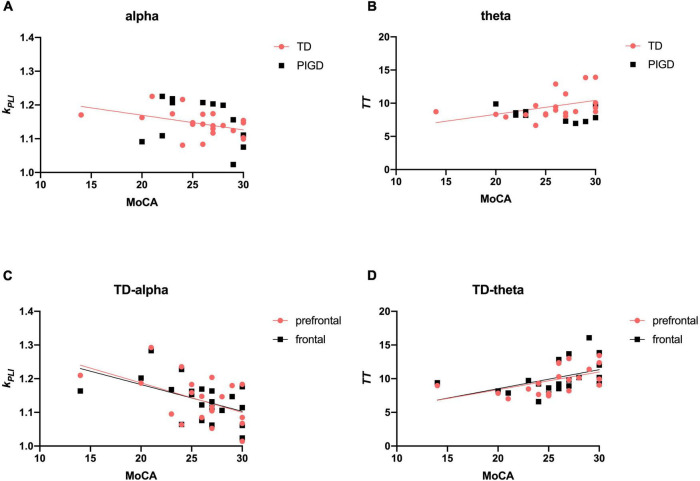
Scatter plot illustrating the correlation between EEG signature and cognition in patients with PD. Upper row: correlations of EEG frequency bands with cognitive data **(A,B)**. Lower row: correlations of EEG characteristic brain regions with cognitive data **(C,D)**. A significant correlation (*p* < 0.05) is marked in solid red and black lines.

We next investigated the relevance between EEG parameters and global cognitive status (MoCA). There were significant direct associations between *k*_*PLI*_ alpha (*r* = −0.466, *p* < 0.05, Figure 3A) and *TT* theta (*r* = 0.571, *p* < 0.01, Figure 3B) with MoCA scores, particularly in the prefrontal and frontal regions (Figures 3C,D).

#### Correlations Between Electroencephalography Signature and Other Non-motor Symptoms

**FIGURE 4:**
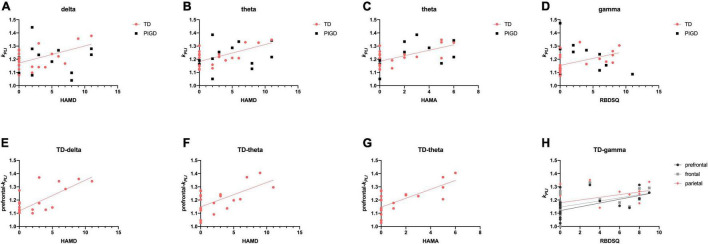
Scatter plot illustrating the correlation between EEG signature and other non-motor symptoms in patients with PD. Upper row: correlations of EEG frequency bands HAMD **(A,B)**, HAMA **(C)**, and RBDSQ **(D)**. Lower row: correlations of EEG characteristic brain regions with HAMD **(E,F)**, HAMA **(G)**, and RBDSQ **(H)**. A significant correlation (*p* < 0.05) is marked in solid red, black, and gray lines.

In the TD group, both *k*_*PLI*_ delta (*r* = 0.539, *p* < 0.05, Figure 4A) and theta (*r* = 0.616, *p* < 0.01, Figure 4B) were positively correlated with increases in HAMD, especially in the prefrontal region (*k*_*PLI*_ delta: *r* = 0.540, *p* < 0.05, Figure 4E; *k*_*PLI*_ theta: *r* = 0.476, *p* < 0.05, Figure 4F). Similarly, a relationship was found between *k*_*PLI*_ theta and HAMA in the prefrontal region (*r* = 0.620, *p* < 0.01, Figures 4C,G). We further analyzed the correlation between EEG and sleep quality (RBDSQ and PDSS-2). *K*_*PLI*_ gamma was particularly associated with RBDSQ scores in the prefrontal (*r* = 0.625, *p* < 0.01), frontal (*r* = 0.609, *p* < 0.01), and parietal (*r* = 0.542, *p* < 0.05, Figures 4D,H) regions in the TD group. No relationship was found between any of the EEG variables with PDSS-2.

### Correlations Between Electroencephalography Characteristic Parameters and Plasma Biomarkers

The relationship between plasma biomarkers and *k*_*PLI*_, *TT*, and *RR* is presented in Tables 3–5, respectively. The level of NfL was negatively correlated with *TT* in the theta band in the TD group (*r* = −0.490, *p* < 0.05, Table 4) and with *RR* in the gamma band in the PIGD group (*r* = −0.727, *p* < 0.01, Table 5). In terms of plasma t-tau, there was a significant positive correlation with *k*_*PLI*_ in the delta (*r* = 0.571, *p* < 0.01), theta (*r* = 0.697, *p* < 0.001), beta (*r* = 0.483, *p* < 0.05), and gamma (*r* = 0.442, *p* < 0.05) frequency bands (Table 3), and a negative correlation with *TT* in the delta band (*r* = −0.552, *p* < 0.05, Table 4) in the TD group. Aβ42 showed an inverse association with *RR* delta in the TD group (*r* = −0.491, *p* < 0.05, Table 5). A close relationship of plasma α-syn with *TT* in the beta frequency band (*r* = 0.983, *p* < 0.001) was found in the PIGD group (Table 4). No statistically significant correlation was found between Aβ42/40 level and EEG measures in patients with PD.

**TABLE 3:** Correlation between plasma biomarkers and *k*_*PLI*_.

Plasma biomarker	Frequency band	TD	PIGD
NfL	Delta	0.207	0.245
	Theta	0.347	0.132
	Alpha	0.333	0.287
	Beta	0.318	0.573
	Gamma	0.187	0.552
T-tau	Delta	0.571^b^	0.098
	Theta	0.697^c^	−0.210
	Alpha	0.100	0.308
	Beta	0.483^a^	0.175
	Gamma	0.442^a^	−0.203
Aβ42	Delta	−0.097	−0.147
	Theta	0.063	−0.275
	Alpha	−0.179	0.119
	Beta	0.201	0.121
	Gamma	−0.167	0.115
Aβ40	Delta	−0.222	−0.089
	Theta	0.008	−0.241
	Alpha	−0.035	−0.101
	Beta	0.218	0.049
	Gamma	0.231	−0.252
Aβ42/40	Delta	−0.227	0.298
	Theta	−0.024	0.475
	Alpha	0.217	0.060
	Beta	−0.301	0.040
	Gamma	−0.253	0.313
α-Syn	Delta	0.093	−0.115
	Theta	0.096	−0.031
	Alpha	0.353	−0.127
	Beta	−0.002	−0.094
	Gamma	−0.062	0.305

*TD, tremor-dominant; PIGD, postural instability and gait disorder; NfL, neurofilament light chain; t-tau, total tau; Aβ42, β-amyloid 42; Aβ40, β-amyloid 40; α-syn, α-synuclein; r, Spearman’s rho.*

*^a^p < 0.05.*

*^b^p < 0.01.*

*^c^p < 0.001.*

**TABLE 4:** Correlation between plasma biomarkers and *TT*.

Plasma biomarker	Frequency band	TD	PIGD
NfL	Delta	0.435	−0.350
	Theta	−0.490^a^	−0.442
	Alpha	0.250	−0.190
	Beta	−0.120	−0.305
	Gamma	−0.183	−0.097
T-tau	Delta	−0.552^a^	−0.168
	Theta	−0.138	−0.277
	Alpha	−0.223	−0.433
	Beta	−0.078	−0.189
	Gamma	0.081	−0.145
Aβ42	Delta	−0.168	−0.147
	Theta	0.050	0.006
	Alpha	−0.134	−0.005
	Beta	−0.044	−0.612
	Gamma	−0.131	−0.146
Aβ40	Delta	−0.018	−0.237
	Theta	0.048	−0.223
	Alpha	−0.161	−0.029
	Beta	−0.007	−0.302
	Gamma	−0.069	0.036
Aβ42/40	Delta	−0.160	0.219
	Theta	−0.147	0.462
	Alpha	0.203	0.084
	Beta	−0.169	−0.325
	Gamma	−0.076	0.026
α-Syn	Delta	0.095	0.013
	Theta	0.059	0.013
	Alpha	0.380	−0.127
	Beta	−0.254	0.983^b^
	Gamma	0.004	0.456

*TD, tremor-dominant; PIGD, postural instability and gait disorder; NfL, neurofilament light chain; t-tau, total tau; Aβ42, β-amyloid 42; Aβ40, β-amyloid 40; α-syn, α-synuclein; r, Spearman’s rho.*

*^a^p < 0.05.*

*^b^p < 0.001.*

**TABLE 5:** Correlation between plasma biomarkers and *RR*.

Plasma biomarker	Frequency band	TD	PIGD
NfL	Delta	0.091	−0.399
	Theta	−0.251	−0.259
	Alpha	−0.013	−0.546
	Beta	0.048	0.228
	Gamma	0.281	−0.727^b^
T-tau	Delta	0.023	−0.064
	Theta	−0.279	0.280
	Alpha	−0.248	0.227
	Beta	0.079	0.362
	Gamma	−0.012	−0.130
Aβ42	Delta	−0.491^a^	−0.021
	Theta	0.099	0.031
	Alpha	0.037	−0.048
	Beta	0.191	0.127
	Gamma	−0.100	−0.390
Aβ40	Delta	−0.222	−0.089
	Theta	0.008	−0.241
	Alpha	−0.035	−0.101
	Beta	0.218	0.049
	Gamma	0.231	−0.252
Aβ42/40	Delta	−0.255	0.328
	Theta	−0.024	0.475
	Alpha	0.217	0.060
	Beta	−0.301	0.040
	Gamma	−0.253	0.313
α-Syn	Delta	0.093	−0.115
	Theta	0.096	−0.031
	Alpha	0.353	−0.127
	Beta	−0.002	−0.094
	Gamma	−0.062	0.305

*TD, tremor-dominant; PIGD, postural instability and gait disorder; NfL, neurofilament light chain; t-tau, total tau; Aβ42, β-amyloid 42; Aβ40, β-amyloid 40; α-syn, α-synuclein; r, Spearman’s rho.*

*^a^p < 0.05.*

*^b^p < 0.01.*

## Discussion

In this study, we aimed to demonstrate the possible association of plasma neurodegenerative proteins and EEG signature with disease progression in early PD with different motor subtypes and explore whether there was a link between plasma biomarkers and EEG DFN signature. For this purpose, we measured several plasma proteins (e.g., NfL, α-syn, t-tau, Aβ42, and Aβ40) quantified by high-sensitivity immunoassays and conducted characteristic DFN analysis for electroencephalographic feature extraction. We performed a correlation analysis between these two kinds of potential markers and a comprehensive clinical assessment battery. Our results suggested that plasma biomarkers and EEG parameters were found to be related to motor severity, cognition, and some NMS symptoms in patients with TD and PIGD. Furthermore, we investigated the correlation between plasma biomarkers and EEG characteristic parameters. Our findings indicated that there was a significant positive correlation between plasma t-tau and *k*_*PLI*_ and pairwise correlations were found among plasma NfL, theta *TT*, and MoCA scores in the TD group. Therefore, plasma biomarkers and EEG measures are considered to be potential tools to predict the disease progression of PD subtypes.

### Plasma Biomarkers and Disease Severity Correlations

As the neural-specific cytoskeletal component and structural component of axon and synapse, NfL plays an essential role in neural electrical signal transmission and posttranslational modification. It is highly expressed in large-caliber myelinated axons and is the main byproduct of neurodegeneration (Yuan et al., 2017). There was a tendency that NfL levels increased as the H&Y stage increased in our PD cohort. No differences were found in the concentrations of NfL between the TD and PIGD groups in our results, consistent with a previous study at baseline (Ng et al., 2020), which suggested that NfL levels were significantly increased in the PIGD group compared with the TD group after a 2-year follow-up, however. Previous studies have reported that NfL levels were significantly elevated in patients with PIGD with worse cognition outcomes and were modestly correlated with MDS-UPDRS III scores, indicating that there was a relationship between NfL and disease severity and progression in PIGD-PD (Ng et al., 2020; Ye et al., 2021). Nevertheless, our results only showed that there was an association between plasma NfL and MDS-UPDRS III, as well as MoCA scores in the TD group, and the correlation was absent in the PIGD cohort. We speculate these conflicting results might be due to the limited number of early-stage patients with PD we enrolled and the lack of follow-up.

It is considered that plasma and cerebrospinal fluid (CSF) amyloid β are reflections of brain amyloidosis (Nakamura et al., 2018; Schindler et al., 2019). Aβ peptides play a crucial role in neuronal information processing and are key components of amyloid plaques deposited in the brains of patients with neurodegenerative diseases, especially common in Alzheimer’s disease and PD dementia (Gomperts et al., 2013). In our cohort, we found the levels of Aβ (Aβ42, Aβ40, and Aβ42/40) were higher in patients with PD than HC, while there was no significant difference between the two motor subtypes, in accordance with another Chinese PD cohort (Ding et al., 2017). Moreover, we are pleasantly surprised that a higher Aβ42/40 level was significantly related to a decrease in HAMD and HAMA in the PIGD group, that is, the lower the Aβ42/40 level is, the greater the possibility of depression and anxiety the patients with PIGD have. Previous studies have confirmed that the plasma Aβ42/40 level was lower in older individuals with depression than in HCs, and Aβ was more inclined to aggregation and polymerization in depression and anxiety patients (Sun et al., 2007; Baba et al., 2012; Nascimento et al., 2015; Johansson et al., 2020). Whether Aβ42/40 level is associated with anxiety and depression emotions in PD subtypes deserves further research.

### Electroencephalographic Features and Disease Severity Correlations

The real relationship between electroencephalographic features and disease severity in patients with PD is not yet definite. To define and quantify brain dynamics and functional connectivity, we conducted a DFN analysis. We extracted three EEG characteristic parameters: *TT*, *RR*, and *k*_*PLI*_. As a symbol of the average duration of the same network state in DFNs, the more the *TT* increased, the longer the same state lasted on the network. *RR* represented the number of times that a network in the same state occurred in DFNs. Both *TT* and *RR* were used to evaluate the degree of network state transition of DFNs (Marwan et al., 2007). In contrast, the *k*_*PLI*_, which was the ratio of standard deviation between fluctuations in real data and fluctuations in alternative data, indicated the magnitude of dynamic fluctuation of brain connectivity. According to our findings, EEG characteristic parameters are proven to have correlations with motor, cognition, and emotions in patients with TD.

Based on our results, *TT* in the delta band was positively correlated with MDS-UPDRS III scores in the TD group, especially in the prefrontal and frontal regions. That means the longer the same state lasts on the network, the worse motor performance the patients with TD have. A possible explanation is that as the motor severity deteriorates, the maintenance of the network state gets longer; that is, the slower the brain network switches. Definitely, the supplementary motor area (SMA), the dorsolateral prefrontal cortex (DLPFC), and the primary motor cortex (M1) are the prime cortical motor regions in the frontal lobe that have been extensively studied. Cortical motor region dysfunction may interpret the pathogenesis of bradykinesia and postural instability and gait disorder, which are the core motor manifestations of PD (Lefaucheur, 2005; Casarotto et al., 2019). This phenomenon is consistent with our findings that delta *TT* in the prefrontal and frontal areas were particularly relevant to motor severity in patients with TD. However, the reason why this correlation disappeared in the PIGD group may be the different mechanisms of altered brain network state in patients with PIGD.

Generally, patients with PD have a slowing tendency of global EEG activity observed to be significantly relevant to cognitive processes, including attention and working memory, executive function, emotion, and so on (Handojoseno et al., 2013). It has already been confirmed that as the cognitive status deteriorates, patients with PD have less stability and functional connectivity of the brain neuronal network, possibly resulting from pathological oscillations and dysregulation in the cortico-basal ganglia-thalamic-cortical pathways and synaptic degeneration (Aarsland et al., 2017; Wang et al., 2020). The frontal and prefrontal cortex assist us to make better decisions. Recent research on clarifying the definite role of neuronal oscillations in the frontal cortex as measured by EEG indicated that frontal theta was an essential integral mechanism in cognitive processes, especially in cognitive control (Cavanagh and Frank, 2014). Similarly, our study showed that *TT* in the theta band was positively correlated with MoCA score in the TD group, particularly in the prefrontal and frontal regions. In addition, alpha *k*_*PLI*_ in the prefrontal and frontal regions were significantly associated with global cognitive function. It could be speculated that the faster the network state switches and the greater the magnitude of dynamic fluctuation of brain connectivity in the frontal cortex is, the worse cognitive function the patients with TD have.

It is widely believed that dopamine (DA) system dysfunction in the substantia nigra pars compacta (SNc) is a trigger for the classical signs and symptoms in patients with PD (Wichmann, 2019). Recently, burgeoning data have connected dopamine system dysfunction to the pathophysiology and pathogenesis of depression and anxiety (Grace, 2016; Calipari, 2020), hence it could be assumed that patients with PD have a greater likelihood of suffering from mood disturbances. A neuroimaging meta-analysis implied increased neural activity and structural changes in the prefrontal regions in depressed and anxious patients with PD (Wen et al., 2016). EEG studies suggested spatiotemporal patterns of cortical activity and functional connectivity altered in patients with PD with affective symptoms (Iyer et al., 2020). Consistent with the above studies, our results suggested a positive correlation between the Hamilton emotion scale with *k*_*PLI*_ in the theta band (especially in the prefrontal region) in patients with TD, further confirming that patients with TD who develop mood disturbances have a greater magnitude of dynamic fluctuation of brain connectivity.

Idiopathic RBD (iRBD) is supposed to be a prodromal stage of PD. Research on resting-state EEG functional connectivity to examine the cerebral network subtle variations in patients with iRBD pointed out that functional networks in iRBD were altered at the early stage of the disease (Sunwoo et al., 2017). *K*_*PLI*_ gamma was particularly associated with RBDSQ scores in the prefrontal, frontal, and parietal regions in the TD group, which have been proved to be decreased regional cerebral blood flow in iRBD in a single-photon emission computerized tomography (SPECT) cerebral blood flow study (Vendette et al., 2011). Accordingly, EEG characteristic parameters show promise to become potential markers for disease progression in patients with TD.

### Correlations Between Electroencephalography Characteristic Parameters and Plasma Biomarkers

In this study, it is the first time to explore the association between EEG features and plasma neurodegenerative proteins in different motor phenotypes of PD. One of the most crucial findings was the relationship of increased *k*_*PLI*_ (higher global dynamic fluctuation magnitude of brain connectivity) with higher t-tau levels in the TD group, which could be speculated that as tau pathology accumulates, so does the discordance of the dynamic brain network. In addition, plasma t-tau and Aβ42 were negatively associated with *TT* and *RR* in the delta band, respectively, which was especially involved in the slow-frequency band, speculating that the more the tau and amyloid pathology deposits in patients with TD, the faster the brain network state switches and the larger fluctuation the brain network has. Similar network alternations associated with biofluid markers have been put forward. For example, increased CSF p- and t-tau levels were correlated with EEG slowing and decreasing synchronization in patients with cognitive impairment (Smailovic et al., 2018; Tanabe et al., 2020); the combined p-tau/Aβ42 ratio exhibited a stronger correlation with the slow-frequency band in elderly individuals (Stomrud et al., 2010). Beyond plasma t-tau, we also found some specific correlations between other plasma biomarkers (NfL and α-syn) and EEG parameters in individual band power. Until now, there is no clear evidence for the link between NfL and EEG variables in PD, and our study first proposed and explored the relationship between them. It is acknowledged that NfL is a byproduct of neurodegeneration, and with the increase in NfL, the severity of neuronal damage aggravates. Our study showed that plasma NfL was negatively correlated with *TT* theta in patients with TD and *RR* gamma in patients with PIGD, speculating that as the plasma NfL increases and the neurodegenerative changes exacerbate, the fluctuation of DFNs becomes wilder. Particularly, pairwise correlations were found among plasma NfL, theta *TT*, and MoCA scores in the TD group, suggesting that an integrated measurement of plasma NfL and theta *TT* may be a powerful predictor of cognitive impairment in patients with TD. Furthermore, it is widely accepted that α-syn accumulation leads to abnormal communication with neuronal, synaptic, and/or dendritic membranes, resulting in pathophysiological changes, which may explain its correlation with network state abnormalities (Caviness et al., 2016). Concurrently, we found α-syn correlated strongly with *TT* beta in patients with PIGD, implying the anomalous network state switching in PIGD. Numerous studies support the hypothesis that positive correlation between blood α-syn and motor severity in patients with PD (Chang et al., 2020; Fan et al., 2020) and our finding that the *TT* in the delta band was positively correlated with MDS-UPDRS III scores and plasma α-syn in the TD group strengthened the hypothesis, implying that motor function impairment might lead to the abnormal slowing of brain state transition. A seemingly contradictory finding was that *TT* was positively correlated with motor impairment and α-syn level while negatively correlated with the levels of NfL and tau. We speculate the probable cause is that the nerve damage characteristics represented by NfL and tau are different from syn since the NFL level mainly reflexes CNS axonal damage severity and tau itself may also have specific CNS damage pathology; that is, the damage of brain functional areas reflected by the accumulation of NfL and tau is different from that of α-syn. Above all, our results support the close relationship between plasma biomarkers and EEG measures. However, the definite correlation between the two kinds of markers and whether quantitative EEG could become an economic and alternative method for the diagnosis and prognosis of PD subtypes require further research and confirmation.

### Limitations

This study has some limitations worth mentioning. First, due to the relatively restricted number of patients and short of EEG conducted among HCs, we cannot infer the definite correlation of EEG characteristic parameters and plasma biomarkers with disease severity in different motor subtypes and compare the discrepancy of EEG variables between PD and controls. Second, it is essential to track the dynamic changes in EEG and plasma protein profiles within individuals and over time to probe whether these could be potential instruments to monitor disease progression. Therefore, much more longitudinal research is badly needed. Finally, the detection of biomarkers in CSF has been extensively studied, which seems to better reflect the function of the central nervous system. In this study, we selected a more convenient blood-based detection means instead of CSF.

## Conclusion

Our study highlighted the reliable relationship of EEG characteristic parameters and plasma biomarkers with disease severity in different motor subtypes of PD and simultaneously explored the potential association between EEG characteristic parameters and plasma biomarkers. Results confirmed that an integrated measurement of plasma NfL and theta *TT* is a powerful predictor of cognitive impairment in patients with TD. As a consequence, these two promising detection methods are expected to become potential markers to predict disease progression of PD subtypes, especially for patients with TD. A combination of these markers for monitoring and prognosis of PD progression deserves further research in larger, follow-up PD cohorts.

## Data Availability Statement

The raw data supporting the conclusions of this article will be made available by the authors, without undue reservation.

## Ethics Statement

The studies involving human participants were reviewed and approved by the Ethics Committee of the General Hospital of Tianjin Medical University. The patients/participants provided their written informed consent to participate in this study.

## Author Contributions

XY and ZeL drafted the manuscript for content, including medical writing for content, the acquisition of data, study concept or design, and analysis and interpretation of data, with contributions from LB, XS, FW, XH, RZ, ZuL, JZ, MD, YW, TC, and SZ. XZ, CL, and CC were responsible for the supervision, project administration, and funding acquisition. All authors read and approved the final version of the manuscript.

## Conflict of Interest

The authors declare that the research was conducted in the absence of any commercial or financial relationships that could be construed as a potential conflict of interest.

## Publisher’s Note

All claims expressed in this article are solely those of the authors and do not necessarily represent those of their affiliated organizations, or those of the publisher, the editors and the reviewers. Any product that may be evaluated in this article, or claim that may be made by its manufacturer, is not guaranteed or endorsed by the publisher.
